# Potassium isotope heterogeneity in the early Solar System controlled by extensive evaporation and partial recondensation

**DOI:** 10.1038/s41467-022-35362-7

**Published:** 2022-12-12

**Authors:** Yan Hu, Frédéric Moynier, Martin Bizzarro

**Affiliations:** 1 Université Paris Cité, Institut de Physique du Globe de Paris, CNRS, UMR 7154, Paris, 75005 France; 2 StarPlan - Centre for Star and Planet Formation, GLOBE Institute, University of Copenhagen, Øster Voldgade 5-7, Copenhagen, DK-1350 Denmark

**Keywords:** Early solar system, Meteoritics, Geochemistry

## Abstract

Volatiles are vital ingredients for a habitable planet. Angrite meteorites sample the most volatile-depleted planetesimal in the Solar System, particularly for the alkali elements. They are prime targets for investigating the formation of volatile-poor rocky planets, yet their exceptionally low volatile content presents a major analytical challenge. Here, we leverage improved sensitivity and precision of K isotopic analysis to constrain the mechanism of extreme K depletion (>99.8%) in angrites. In contrast with the isotopically heavy Moon and Vesta, we find that angrites are strikingly depleted in the heavier K isotopes, which is best explained by partial recondensation of vaporized K following extensive evaporation on the angrite parent body (APB) during magma-ocean stage. Therefore, the APB may provide a rare example of isotope fractionation controlled by condensation, rather than evaporation, at a planetary scale. Furthermore, nebula-wide K isotopic variations primarily reflect volatility-driven fractionations instead of presolar nucleosynthetic heterogeneity proposed previously.

## Abstract (web summary)

This study reports strikingly light K isotopic compositions for the extremely K-depleted angrite meteorites, thereby providing the first observation of isotope fractionation likely controlled by partial recondensation at a planetary scale.

## Introduction

Condensation and evaporation play a central role in shaping the volatile inventory of planets and their potential for harboring life. The inner Solar System, which features terrestrial planets, the Moon, and the asteroid belt, is depleted in volatile elements relative to the bulk solar composition sampled by carbonaceous Ivuna-type (CI) chondrites. This depletion has been debated to reflect either 1) devolatilization of interstellar dust and inheritance of the depletion by the inner solar nebula^1^, 2) incomplete condensation from the dispersing solar nebula^2^, 3) accretion of volatile-depleted materials like chondrules^3,4^, or 4) evaporative loss during planetary accretion or degassing^5–7^. Studies of moderately volatile elements (MVEs) have provided valuable insights to distinguish between these scenarios because their isotopes are fractionated to different extents and/or in different directions during these processes. Here we use stable K isotopes to study volatile depletion processes in planetary bodies. Potassium is among the most abundant MVEs, and K/U ratios measured in meteorites provide a convenient assessment of the relative depletion of MVEs to refractory elements in their parent bodies^6–8^.

Compared with CI chondrites, K is highly depleted in terrestrial bodies, with the Moon and Vesta showing similarly high levels of depletion (~ 95%), followed by Earth (86%) and Mars (76%)^6–8^. This extensive depletion has motivated the search for K isotope fractionation associated with evaporation. However, pioneering analyses by Humayun and Clayton^9^ on a wide variety of planetary materials yielded indistinguishable ^41^K/^39^K ratios within ±0.5‰, which is inconsistent with significant isotope fractionation predicted by free evaporation. These results suggest suppressed isotope fractionation (sub-permil) under high vapor pressure conditions^10^, as postulated for Moon formation from a thick gas envelope after the Giant Impact^11^. Recent advances in analytical precision have revealed increasing depletion of light K isotopes on Earth, Mars, the Moon, and Vesta with decreasing planetary sizes, which is consistent with bulk K depletion in these celestial bodies^12^. This correlation suggests that the final volatile content of a planetary body is intrinsically governed by its size, with smaller bodies having lower escape velocities that facilitate the evaporative loss of silicate vapors^6,12^. A critical test for this universal size control on the volatile budget of a terrestrial body is the angrite parent body (APB), which represents the depletion endmember of MVEs in the Solar System^6–8^ and is the focus of this study.

Angrites, named after the witnessed fall of Angra dos Reis (hereafter AdoR), are a rare group of achondrites currently consisting of 22 unpaired samples. They figure prominently in constraining the volatile history of the early Solar System because of their antique age, pristine composition, and pronounced volatile depletion^13–16^. Angrites are among the oldest dated meteorites^17^, formed as early as 4 Myr after the condensation of calcium-aluminum-rich inclusions (CAIs)^18,19^. Most angrites have preserved their original texture and composition due to a lack of shock- or impact-induced metamorphism and brecciation that occurred pervasively in other basaltic achondrites^20^. Compositionally, angrites are unique among achondrites in displaying small but systematic super-chondritic isotope ratios for major planet-forming elements (Mg, Si, and Fe) that are normally difficult to vaporize^5,21–23^. The extent to which these isotopic signatures reflect volatile depletion or planetary differentiation remains controversial. Unlike Mg, Si, and Fe, K is a highly incompatible lithophile element such that its isotopes do not fractionate by core formation or basaltic differentiation^24,25^. Furthermore, the moderately volatile K is most severely depleted in angrites (99.8%) among igneous meteorites^6–8^. Therefore, significant K isotope fractionations are expected for angrites, but the analyses are challenged by their low K abundances.

Here, we utilize the latest Nu Sapphire^TM^ collision-cell multi-collector inductively-coupled plasma mass spectrometer (CC-MC-ICP-MS) to analyze K isotopic compositions in representative aliquots of angrites and chondrites, complemented with elemental concentration measurements. The improved collision-cell system of Sapphire^TM^ reduces argon-related isobaric interferences to negligible levels. This new design eliminates the need to narrow the ion beams to achieve high mass resolution, leading to a considerable increase in K sensitivity (>2000 V/ppm) relative to conventional instruments (typically < 10 V/ppm)^26^. It thus provides an opportunity to investigate the extremely K-depleted rocks and associated K isotope fractionation. We find that angrites have strikingly light K isotopic compositions, most likely resulting from incipient recondensation following extensive K evaporation. The proposed planetary-scale isotope fractionation by condensation on the APB, where volatile depletion has proceeded to extremes, contrasts with the dominant control of evaporation on planetesimals with less severe volatile depletion (e.g., Vesta and the Moon)^12^. In both cases, K isotopic variability among planetary bodies suggests volatility-driven fractionation rather than a heterogeneous distribution of presolar nucleosynthetic components.

## Results and discussion

### Potassium isotopic compositions of chondrites and terrestrial standards

Potassium isotopic data are reported in Tables 1 and 2. Analytical accuracy was verified by measurements on well-characterized terrestrial standards and witnessed chondrite falls (Supplementary Fig. 1). Seawater is the isotopically heaviest terrestrial reservoir and has a δ^41^K value of 0.14 ± 0.02‰, which is higher than all meteorites analyzed in this study. Seven analyses of Orgueil (CI1) give an average δ^41^K of −0.10 ± 0.02‰, consistent with published results (−0.24 ± 0.03‰ to −0.04 ± 0.04‰)^27–29^. Ornans (CO3) is isotopically similar to Orgueil, and its δ^41^K value (−0.08 ± 0.05‰) is within the range reported for other nine CO chondrites (−0.35 ± 0.07‰ to 0.09 ± 0.03‰)^27,28,30,31^. Murchison (CM2) and Allende (CV3) show significant isotopic variations from −0.41 ± 0.05‰ to −0.10 ± 0.03‰ and −0.62 ± 0.04‰ to −0.08 ± 0.03‰, respectively^27,28,30,31^. Our results for Murchison (−0.24 ± 0.04‰) and Allende (−0.14 ± 0.03‰) fall within these ranges. Abee (EH4) has a δ^41^K value (−0.35 ± 0.05‰) comparable to terrestrial basalts, such as BCR-2 (−0.43 ± 0.03‰). Two ordinary chondrites, Kernouve (H6) and Tuxtuac (LL5), have the lowest δ^41^K values of −0.67 ± 0.05‰ and −0.56 ± 0.04‰, respectively.

**Table 1:** Potassium isotopic compositions of terrestrial samples and chondrites analyzed in this study

Sample	Description	Type	δ^41^K (‰)	2 SD (‰)	95% c.i. (‰)	N
**Terrestrial samples**						
Seawater			0.13	0.03	0.03	6
Duplicate			0.16	0.08	0.02	7
Duplicate			0.14	0.07	0.03	7
Duplicate			0.15	0.04	0.03	7
Wtd average			0.14		0.02	
BCR-2	Basalt		−0.43	0.05	0.03	8
**Chondrite samples**						
Orgueil-1	Carbonaceous	CI1	−0.13	0.03	0.03	7
Duplicate			−0.09	0.05	0.06	6
Duplicate			−0.09	0.04	0.04	7
Duplicate			−0.10	0.06	0.04	6
Duplicate			−0.11	0.04	0.05	7
Orgueil-2			−0.08	0.06	0.05	6
Duplicate			−0.10	0.04	0.04	6
Wtd average			−0.10		0.02	
Murchison	Carbonaceous	CM2	−0.23	0.06	0.02	8
Replicate			−0.26	0.07	0.02	8
Wtd average			−0.24		0.04	
Ornans	Carbonaceous	CO3	−0.08	0.09	0.05	6
Allende	Carbonaceous	CV3	−0.14	0.03	0.02	7
Duplicate			−0.13	0.10	0.04	7
Wtd average			−0.14		0.03	
Abee	Enstatite	EH4	−0.35	0.09	0.05	7
Kernouve	Ordinary	H6	−0.67	0.08	0.05	7
Tuxtuac	Ordinary	LL5	−0.56	0.03	0.04	7

Tables 1 and 2 present the δ^41^K values (‰), two standard deviations (2 SD), and 95% confidence interval (c.i.) for a sample solution measured N times during an analytical session. The 95% c.i. is calculated from t × [SD/√(N-1)], where t is the Student’s *t* number and SD is the standard deviation of δ^41^K values calculated for all measurements of bracketing standard (NIST 3141a) during a session.

Duplicate represents repeat instrumental analysis on the same purified K solution during different sessions.

Replicate represents repeat column chemistry from the same bulk dissolution.

The labels ‘−1’ and ‘−2’ attached to the name of a given sample refer to a new dissolution, column chemistry, and instrumental analysis.

Wtd average is the error-weighted average.

**Table 2:** Potassium concentrations and isotopic compositions of angrites analyzed in this study

Sample	[K]	δ^41^K	2 SD	95% c.i.	N	Meteorite mass	Dissolved mass
	(ppm)	(‰)	(‰)	(‰)		(g)	(mg)
NWA 7203	36.7	−0.74	0.03	0.03	6	107	82.8
NWA 12320	67.8	−0.36	0.06	0.03	6	4460	124.2
Sah 99555-1	45.3	−0.56	0.02	0.03	6	2710	125.3
Sah 99555-2		−0.57	0.04	0.03	6		133.9
Wtd average		−0.56		0.02			
NWA 12774	16.4	−1.18	0.03	0.03	6	454	127.2
NWA 12004-1	109.1	−0.41	0.06	0.04	7	183	123.8
Duplicate		−0.43	0.03	0.02	7		
NWA 12004-2		−0.46	0.06	0.05	6		62.5
Duplicate		−0.45	0.05	0.04	6		
Wtd average		−0.44		0.02			

### Potassium isotopic compositions of angrites

Angrites were found primarily in deserts, and the only witnessed fall, AdoR, is not readily available. To mitigate the influence of terrestrial alteration and magmatic differentiation on the chemical and isotopic compositions of angrites, five out of the eight relatively massive (>100 g) volcanic angrites were selected: NWA 7203, NWA 12004, NWA 12320, NWA 12774, and Sahara (Sah) 99555 (Supplementary Note 1). Chemical weathering appears to be limited in these samples, in contrast to the positive Ce anomaly identified in the small (46.2 g) angrite NWA 7812 (Supplementary Fig. 2a). Furthermore, volcanic angrites cooled rapidly after the eruption (10–50 °C/hour)^15^, as evidenced by their quenched texture. Therefore, limited crystal fractionation occurred, and representative bulk compositions can be obtained with relatively small sample sizes, which contrasts with plutonic angrites that underwent more extensive fractional crystallization (Supplementary Fig. 2)^19^. Volcanic angrites also have smaller cosmic ray exposure (CRE) ages^32^, hence are less affected by the cosmogenic production of ^41^K from ^41^Ca. The selected samples encompass the compositional range documented for volcanic angrites (Supplementary Tables 1 and 2 and Supplementary Fig. 2) and provide a reasonably representative sampling of the interior of the APB from where they originated.

**Fig. 1:**
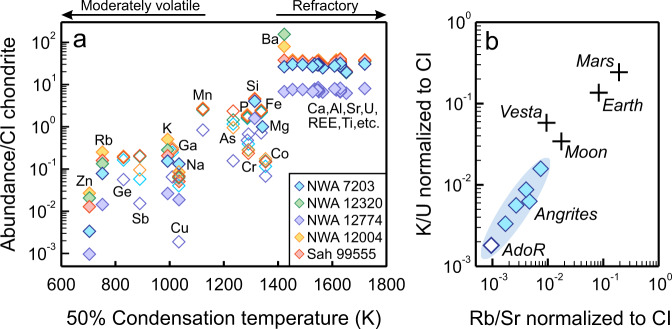
Extensive depletion of moderately volatile elements (MVEs) in angrites. **a** Elemental concentrations of angrites analyzed in this study are plotted relative to concentrations in CI chondrites^45^ (both are normalized to Mg = 1.0) versus 50% nebular condensation temperatures^57^. Lithophile elements are shown in solid symbols and siderophile elements are shown in open symbols. REE refers to rare earth elements. **b** Angrites, as indicated by the blue field, represent the endmember for MVE depletion among planetary basalts. The degrees of volatile depletion in AdoR, the bulk silicate Moon, Vesta, Earth, and Mars are assessed by CI-normalized Rb/Sr and K/U ratios from ref. 8.

**Fig. 2:**
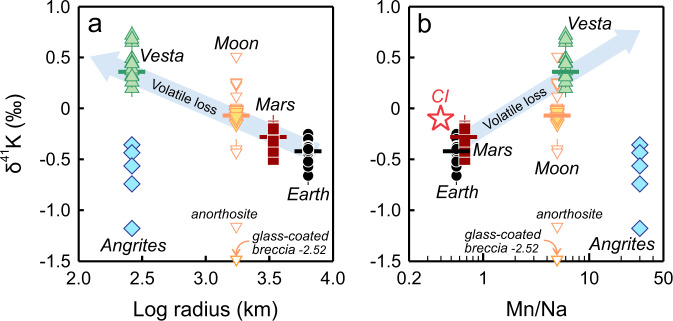
Angrites deviate from the trend of increasing δ^41^K with enhanced volatile depletion defined by other planetary basalts. The blue arrows illustrate the direction of increasing volatile loss indicated by **a** smaller planetary size and **b** elevated Mn/Na ratio. The δ^41^K values of differentiated terrestrial bodies are from the literature, including Earth (refs. 24, 25, 66), Mars (refs. 12, 27, 34), the Moon (refs. 11, 33), and Vesta (refs. 27, 34). The Mn/Na ratios of CI chondrites, Earth, and Mars are from refs. 45, 67, and 4, respectively, and those of the Moon, Vesta, and angrites are from ref. 7. The colored horizontal bars represent average δ^41^K values. For lunar rocks, filled symbols are mare basalts, and open symbols are non-mare rocks. δ^41^K error bars are plotted as 95% c.i. When not seen, they are smaller than the sample symbols.

The five samples studied consist primarily of Al, Ti-rich clinopyroxene, Ca-bearing olivine, and nearly pure anorthite (An ≥ 99.4), which typically occur in subequal modal abundances in angrites (Supplementary Note 1 and Supplementary Fig. 3). There is widespread depletion of MVEs in these samples (Fig. 1a), and the depletion is more pronounced than in other rocky bodies (Fig. 1b). Nevertheless, these samples contain higher alkali abundances than AdoR with variable concentrations of Na (97.4 to 274.0 ppm), K (16.4 to 109.1 ppm), and Rb (0.04 to 0.23 ppm). Unexpectedly, while angrites represent the most K-depleted planetary basalts, they are isotopically the lightest and deviate from the trend of increasing δ^41^K with enhanced volatile depletion related to smaller planetary size or elevated Mn/Na ratio (Fig. 2). Their δ^41^K values vary substantially from −1.18 ± 0.03‰ to −0.36 ± 0.03‰, which are distinctly lower than those of Mars (−0.28 ± 0.18‰^12^), the Moon (−0.07 ± 0.09‰^11,33^), and Vesta (0.36 ± 0.16‰^34^). The highest δ^41^K in angrites is comparable to that of the bulk silicate Earth (−0.42 ± 0.07‰^24^), whilst the lowest δ^41^K resembles that of a lunar anorthosite 60015 (−1.16 ± 0.04‰)^33^, which is the isotopically lightest lunar sample reported except for a glass-coated breccia 64435 (−2.52 ± 0.08‰)^33^.

The finding that angrites are isotopically lighter than Vesta by 1.6‰ is inconsistent with the previous suggestion that K isotopic compositions of planetary bodies scale negatively with their sizes^12^. Although the APB has not been physically identified, it is inferred to be comparable in size to Vesta (~ 262.7 km radius^35^). A minimum radius of 100 km is required for the APB to have sufficient surface gravity to retain pyroclastically erupted basalts in the crust^36^. This estimate is consistent with the threshold size for sustaining a short-lived core dynamo on the APB, as manifested by the thermoremanent magnetization in angrites^37^. The size of the APB has also been estimated from the solubility of water and carbon in primitive angrite melts, which requires a confining pressure higher than 166 MPa, corresponding to a radius of 270 km to possibly 340 km^38^. Despite the possibly similar size of the APB and Vesta, their contrasting δ^41^K signatures suggest that factors other than size are equally important in controlling the δ^41^K values of angrites, which are discussed below.

### Assessing cosmogenic effects in angrites

**Fig. 3:**
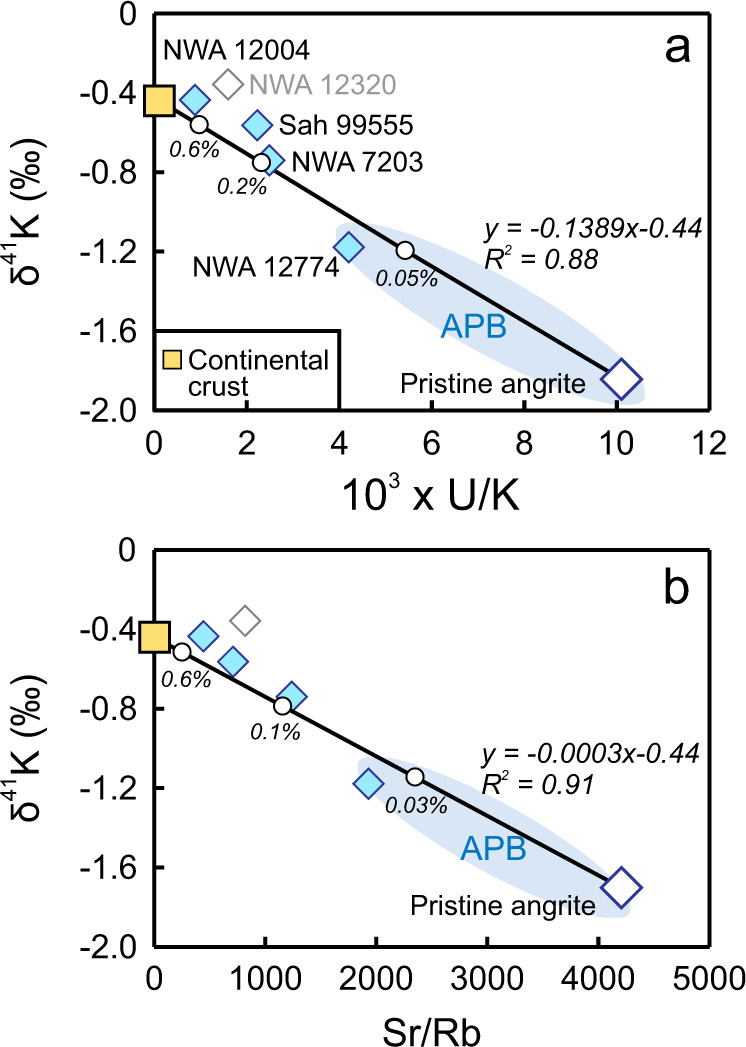
Negative correlations of δ^41^K values with U/K ratios and Sr/Rb ratios. The coherent correlations in **a** and **b** most likely reflect contamination of the pristine angrite composition (low in δ^41^K, K, and Rb) by a crustal component with higher δ^41^K, K, and Rb. The yellow square denotes the average composition of the upper continental crust, with 2.8 wt% K_2_O and 84 ppm Rb (ref. 49), and a δ^41^K value of −0.44 ± 0.05‰ (ref. 50.). The δ^41^ K value of pristine angrite is extrapolated from the linear regression of measured angrites and upper continental crust, and is shown by the open diamond symbol with dark blue border. The blue fields indicate the δ^41^K range inferred for the angrite parent body (APB). The numbers along the binary mixing lines in black indicate the fraction of crustal contamination. Sample NWA 12320, shown by an open symbol with grey border, is not included in the linear regression as it is likely unrepresentative of the bulk composition of this angrite due to the presence of secondary barites formed during aqueous alteration. Please refer to Supplementary Fig. 5 for more detailed discussions. δ^41^K error bars (95% c.i.) are smaller than the sample symbols.

Given that K isotope fractionation during mantle melting and basaltic differentiation is negligible^24,25^, δ^41^K values measured in angrites reflect those of the APB with varying degrees of post-eruption modification. The abundance of ^41^K in angrites could be affected by radiogenic ingrowth from the decay of short-lived ^41^Ca (half-life = 0.1 Myr), resulting in a coupled increase in δ^41^K and K concentration. The K concentration in angrites would also increase with basaltic differentiation, but this process does not affect the U/K ratio given the comparably low solid/melt partition coefficients of U and K. Furthermore, the measured U concentrations in the five studied angrites (0.07 to 0.11 ppm) agree with those calculated (0.07 to 0.11 ppm) from their respective Sr concentrations assuming a CI-like U/Sr ratio. This agreement suggests no significant remobilization of U during their residence in deserts. Therefore, variations in their U/K ratios primarily reflect post-eruption additions of K. The inverse correlation between δ^41^K and U/K (Fig. 3a) shows that δ^41^K value and K concentration increased concomitantly, which could be due to cosmogenic enrichment or terrestrial alteration.

Accumulation of cosmogenic ^41^K in angrites depends on their CRE ages and Ca/K ratios. Angrites with higher Ca contents have more ^40^Ca to produce ^41^Ca via a thermal neutron capture reaction ^40^Ca(n, γ)^41^Ca, and lower K contents minimize dilution of cosmogenic ^41^K by indigenous K. Therefore, angrites with greater CRE ages and higher Ca/K ratios would be expected to have higher δ^41^K values. The cosmogenic effects are evaluated using the two angrites for which CRE ages are available. NWA 7203 has a greater CRE age (20.3 Myr vs. 6.8 Myr^39,40^) and a higher Ca/K ratio (3006 vs. 2304) than Sah 99555, but a lower δ^41^K value (−0.74 ± 0.03‰ vs. −0.56 ± 0.02‰), contrary to the expectation from cosmogenic effects. Unlike K, Rb would not be affected by the decay of ^41^Ca. Nevertheless, the negative correlation between δ^41^K and Sr/Rb suggests that δ^41^K values increase with Rb concentrations in a similar manner as with K concentrations (Fig. 3b). Therefore, these coherent trends do not reflect cosmogenic effects but require a common chemical processing of alkali elements in angrites, which is most consistent with terrestrial contamination.

### Constraining pristine K/U ratios in angrites

As the studied angrites were collected in the Sahara Desert, it is critical to evaluate the effects of terrestrial alteration. This evaluation requires a pristine angrite composition for reference. AdoR was collected shortly after falling to Earth; hence, it has the least terrestrial influence^8^. It is also notable for being composed dominantly of augite (93 vol.%), and its origin as magmatic cumulate or porphyritic igneous rock remains debated^15^. Nevertheless, AdoR shares the same Δ^17^O and nucleosynthetic ε^54^Cr anomalies with other angrites^18,41^, implying that they likely originated from the same parent body. Furthermore, both volcanic and plutonic angrites, including AdoR, plot along the 4.56 Ga isochron of ^87^Sr/^86^Sr vs. ^87^Rb/^86^Sr^14^, indicating their sources underwent a single episode of fractionation in Rb/Sr, and by inference, K/U. The two groups of angrites also define a single ^53^Mn-^53^Cr isochron, suggesting their sources diverged from chondritic evolution concurrently due to a planetesimal-wide differentiation^18^. Regardless of the specific origin of AdoR and its distinct petrology, it remains the best representative of the pristine K/U (and Rb/Sr) signature of the APB, given the similar incompatibility between K and U (and between Rb and Sr).

The K and Rb concentrations of AdoR are evaluated by comparing literature data with extrapolated results and with back-calculated K abundances from radiogenic ^40^Ar data. The isotope dilution method presumably provided the most reliable analyses of AdoR, which returned 12.9 ppm K and 0.0311 ppm Rb^42^. These values agree well with the 13.2 ppm K and 0.0318 ppm Rb extrapolated from the average K/Rb (415), Rb/Sr (0.0002377), and Sr concentration (134 ppm) compiled for AdoR (Supplementary Note 2, Supplementary Table 3, and Supplementary Fig. 4). This K concentration is also consistent with the 6.4 to 11 ppm K (Supplementary Table 4) required to bring the K-Ar and U/Th-He ages into agreement (~ 4.5 Ga)^43^. In comparison, Wasserburg et al^44^. obtained a slightly higher K concentration (32 ppm) for AdoR, which corresponds to a considerably younger K-Ar age (2.8 Ga) and is not considered further. Assuming a CI-like Sr/U ratio (1004, ref. 45.) for AdoR, its U concentration is calculated to be 0.133 ppm, yielding a K/U ratio of 99.

The K/U ratios of other angrites are calculated to check if they agree with that of AdoR. Noble gas analyses in four angrites revealed broadly coincident U/Th-He ages of 4.2 to 4.6 Ga^40,43^. By fitting their K-Ar ages to 4.5 Ga, the calculated K concentrations converge to a narrow range averaging at ~ 10 ppm despite wide-ranging concentrations measured up to 350 ppm (Supplementary Table 4). Independently, pristine K concentrations in angrites can be reconstructed from mineral data, assuming that terrestrial contamination is concentrated on angrite surface and between mineral grain boundaries. The average K concentrations for plagioclase (20 ppm), clinopyroxene (7 ppm), and olivine (3 ppm) are derived from mineral compositions of five representative angrites^46^. These minerals typically occur in similar modal abundances in angrites (Supplementary Fig. 3), resulting in a bulk K concentration of 10 ppm, which agrees with that derived from ^40^Ar. With an average U concentration of 0.1 ppm calculated from 14 basaltic angrites (Supplementary Table 5), a K/U ratio of 100 is obtained. The similar K/U ratios in AdoR and other angrites indicate a homogeneous K/U ratio in their mantle sources and possibly the APB as a whole.

### Evaluating terrestrial effects on angrites

Our angrites have variably higher K/U ratios than AdoR (Fig. 1b), which contradicts the effects of K leaching by chemical weathering and suggests K additions due to crustal contamination. Significant leaching of K is prevented by limited precipitation in hot deserts and the lack of brecciation or shock-induced microfractures in angrites for pervasive fluid percolation^20,47^. This inference is supported by the absence of clay minerals in our samples, which typically form during feldspar hydrolysis. Notably, NWA 12774, the sample with the lowest δ^41^K, is characterized by a very fresh interior. In addition, the total major oxide contents of these angrites range from 99.3 to 100.4 wt.% (Supplementary Table 1), suggesting no appreciable leaching of cations that are more labile than K (e.g., Mg^2+^ and Fe^2+^). Moreover, none of the angrites shows fractionation of oxidized, less soluble Ce^4+^ from trivalent rare earth elements (Supplementary Fig. 2a), as is often observed in weathered eucrites from Antarctica where meltwater is available^48^. The only chemical feature possibly related to desert weathering is the elevated Ba concentration and Ba/La ratio in NWA 12320 relative to other angrites (Supplementary Fig. 5), suggesting the presence of secondary barites due to exposure to isotopically heavy salty fluids. Therefore, the slightly higher δ^41^K of NWA 12320 may be related to aqueous alteration, whereas the variably lower δ^41^K in other angrites require a different process.

Crustal addition of alkalies to angrites is evident from the ubiquitously disturbed K-Ar and Rb-Sr dating results^14,16,40^. Compared with AdoR^42^, the upper continental crust (UCC) is enriched in K and Rb by a factor of ~1800 and 2700, respectively^49^. Hence even minor contamination would have a significant effect on angrites. The largely linear correlation between δ^41^K and U/K (Fig. 3a) suggests mixing between two compositionally uniform but distinct components. Furthermore, the K-rich contaminant is characterized by a low U/K ratio and a high δ^41^K value. In the UCC, shales that are composed mainly of clay minerals vary in δ^41^K from −0.68 to −0.12‰ due to prolonged fluid-rock interaction^50^. By contrast, loess sediments from five geographic provenances worldwide show limited variation (−0.47 to −0.35‰), consistent with their less weathered nature^50^. These silt-sized sediments are more representative of the aeolian dust that covers the Sahara Desert. The δ^41^K values of losses (−0.42 ± 0.07‰, 2 SD) also agree with the weighted average of the UCC ( − 0.44 ± 0.05‰, 2 SD)^50^. Because the latter value is based on a larger dataset, it is considered more representative of the crustal component that contaminated the studied angrites.

### Extrapolating the δ^41^K value of the angrite parent body

Angrites are randomly sampled fragments of the APB over a wide time window of ~ 56 Myr^32^, and they likely originated from various locations within the APB. The binary mixing line between δ^41^K and U/K in angrites (Fig. 3a) indicates that their mantle sources share a homogeneous K isotopic composition. This contention is supported by the uniform Δ^17^O and ^54^Cr/^52^Cr in angrites, which cannot be achieved at subsolidus temperatures due to sluggish diffusion but requires a global-scale melting^18,41^. The resultant magma ocean and mantle convection should have also homogenized the distribution of K isotopes. Although petrologic studies^51^ and Hf-W isotopic data^52^ suggest that the APB mantle may contain variable proportions of olivine, spinel, and residual metal, none of them is a major carrier of K; therefore, local-scale heterogeneity in mineral distribution would not affect a global homogeneity in δ^41^K.

The δ^41^K value of pristine angrites, and hence the APB, can be extrapolated based on the mixing relationships shown in Fig. 3, which yields a δ^41^K value of −1.84‰ and −1.70‰ from the pristine U/K and Sr/Rb ratios estimated for the APB, respectively. The consistent estimates from U/K and Sr/Rb ratios further substantiate the conclusion that the K isotopic variations in angrites reflect variable levels (0.05–0.6%) of crustal contamination. In addition to the pristine δ^41^K extrapolated for the APB, the value of NWA 12774 (−1.18‰) provides an upper bound for the APB, as it is the least contaminated angrite in our sample suite. Accordingly, the K isotopic composition of the APB is inferred to fall between −1.84‰ and −1.18‰. Regardless of the uncertainty in the extrapolation, the APB is distinguished from other terrestrial bodies by its strikingly light K isotopic composition, which could be either an inherited feature from the APB precursor or a later-stage, process-driven signature.

### Origin of extreme K depletion on the angrite parent body

Numerical simulations of planet formation show that planetary embryos grow more rapidly via gas drag-assisted preferential accretion of chondrule-like pebbles than by collisional accretion of planetesimals^53^. Chondrules are the primary carrier of volatile depletion in chondrites^3^. If the extreme alkali deletion on the APB reflects the nature of a chondrule-like precursor, Rb depletion should precede accretion and differentiation of the APB. The depletion of MVEs on the APB occurred shortly after CAI formation (0.23–1.8 Myr), as evidenced by the indistinguishably low initial ^87^Sr/^86^Sr in angrites and CAIs^14,16^. Nevertheless, this timescale does not definitively predate the accretion of the APB and its subsequent differentiation driven by radiogenic heating from the decay of short-lived ^26^Al (t_1/2_ = 0.73 Myr) (Supplementary Fig. 6). The initial ^26^Al abundance constrained for the APB is four times lower than the canonical CAI value, requiring the APB to have accreted within 0.25 ± 0.15 Myr of CAI formation to permit a global-scale melting initiated 0.40 Myr after CAI formation^54^. This early accretion of the APB is compatible with its core-mantle differentiation at 1.9 ± 0.8 Myr based on ^182^Hf-^182^W systematics^52^. The rapid accretion and differentiation of the APB render ambiguities in distinguishing the volatile depletion mechanism on the APB based on Rb-Sr dating.

**Fig. 4:**
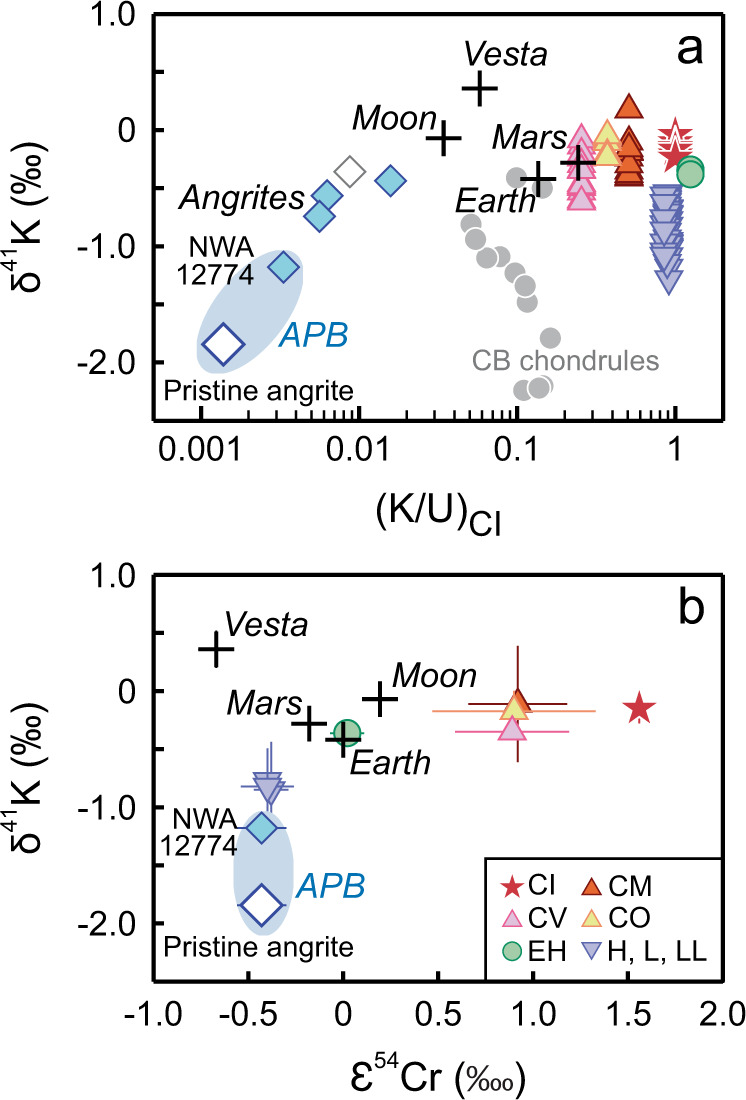
Potassium isotopic compositions of the angrite parent body (APB) compared with other planetary materials. In **a** the extremely low K/U ratio and δ^41^K value of the APB do not resemble any of the known planetary materials; therefore, these characteristics are not inherited from the precursor of APB. In **b**, the widely divergent δ^41^K signatures of the APB and Vesta, despite their similar nucleosynthetic ε^54^Cr anomalies, suggest that δ^41^K values are controlled by volatility-dependent fractionations rather than nucleosynthetic anomalies. The plotted δ^41^K values for chondrite witnessed falls and chondrules include data from this study and those from the literature (refs. 27–31, 68). Individual data are plotted in **a**. Error bars (95% c.i.) are typically similar to or smaller than the sample symbols and are omitted for clarity. Group averages are plotted in **b** with associated 2 SD. The δ^41^K values of differentiated planetary bodies are also plotted for comparison (refs. 11, 12, 24, 33, 34). The K/U ratios are from ref. 8., except those for angrites (from this study) and those for CB chondrules (from ref. 29). The average nucleosynthetic ε^54^Cr anomalies and associated 2 SD are from refs. 56, 69. The blue fields indicate the δ^41^K range inferred for the APB, which is bounded by the extrapolated value for pristine angrite (shown in open diamond symbol with dark blue border) and the isotopically lightest angrite measured in this study (NWA 12774). The open diamond symbol with grey border denotes angrite NWA 12320 that might be affected by aqueous alteration.

The strikingly low δ^41^K values and K/U ratios characteristic of angrites do not resemble any documented chondrite compositions (Fig. 4a). Therefore, it seems unlikely that these features were inherited from the APB precursor. Even chondrules contain substantially higher K concentrations than angrites. Chondrules account for ~ 85 vol.% of the ordinary chondrites^3^, which have CI-like, high K/U ratios (Fig. 4a), although their δ^41^K values and nucleosynthetic ε^54^Cr anomalies are closest to the APB (Fig. 4b). In comparison, chondrules in carbonaceous chondrites have been estimated to contain 260 ± 14 ppm K with an average δ^41^K value of −0.33 ± 0.12‰^28^, which is isotopically much heavier than that inferred for the APB. Lower δ^41^K values (down to −2.24‰) have been reported recently for CB chondrules (Fig. 4a), interpreted to reflect incomplete condensation from an impact-generated vapor plume^29^. The CB chondrules formed much later (~4.8 Myr after CAI formation, ref. 55) than the APB and have a positive ε^54^Cr anomaly (1.36 ± 0.30, ref. 56) that contrasts with the negative anomaly of the APB ( − 0.42 ± 0.13, ref. 18). Therefore, a direct genetic link between the two is unlikely. Nonetheless, given that condensation occurs more readily on larger bodies than on millimeter-sized chondrules^10^, the APB may have also acquired its low δ^41^K signature via a vapor condensation process.

The chemical and isotopic compositions of angrites support heat-driven devolatilization during planetary evolution. Most notably, the APB is characterized by a super-chondritic Mn/Na ratio that indicates more severe depletion of Na than Mn (Fig. 2b). This contrasts with the similar volatilities of Mn (T_50_ = 1123 K) and Na (T_50_ = 1035 K) under nebular conditions that are H_2_-rich and low in oxygen fugacity (log*f*O_2_ = IW-7, where IW indicates the iron-wüstite buffer)^57^. In comparison, angrites formed at much higher oxygen fugacity (log*f*O_2_ = IW + 1)^15^. Therefore, elevated Mn/Na ratio in the APB cannot be explained by volatile depletion under reducing nebular conditions. Instead, it is more consistent with silicate evaporation under oxidizing planetary conditions, during which the monovalent Na is more volatile than the divalent Mn^7,58^. Post-nebular volatile depletion is also manifested by the chondritic Fe/Mn ratios in angrites, which reflect a balance between Fe depletion by core formation and a greater loss of Mn over Fe during planetary devolatilization^59^. Furthermore, angrites have the highest δ^57^Fe, δ^30^Si, and δ^26^Mg values among planetary basalts^5,21–23^, which is most consistent with global-scale evaporation and preferential loss of light isotopes to silicate vapors.

To reconcile the light K isotopic composition of the APB with its heavy isotopic compositions of Si, Fe, and Mg, it is most likely that during the partial evaporation of Si, Fe, and Mg, the more volatile K has been quasi-entirely vaporized. A small fraction of the vaporized K condensed back onto the APB as it cooled down. The possibility of complete K evaporation on the APB can be assessed by comparing the extent of volatile depletion on the APB and Vesta (Supplementary Fig. 7). Based on the Fe/Mn ratio and δ^57^Fe value, the APB is estimated to have lost ~20% and ~80% of its initial Fe and Mn budgets, respectively^59^. In contrast, Vesta preserves a chondritic δ^57^Fe, suggesting that it has retained the nebular Fe and Mn inventories^59^ despite considerable depletions in K (94.2%) and Rb (99.0%)^8^. Given that the APB is depleted in Mn by 80% relative to Vesta, it is reasonable to assume that K and Rb have been completely evaporated from the APB. Therefore, the vanishingly small amounts of K and Rb measured in angrites represent incipient condensation on the APB, whereas the budget of more refractory elements (e.g., Mg, Fe, Si) represents evaporative residuum.

Planetesimal-wide evaporation on the APB probably occurred during the global magma-ocean stage, given the overlapping timescales of Rb depletion^14,16^ and core-mantle differentiation^52^ (Supplementary Fig. 6). As heating from the decay of ^26^Al became trivial ~2 Myr after CAI formation^54^, subsequent cooling of the APB led to a decrease in equilibrium vapor pressure, thereby oversaturation of the vaporized K in the surrounding vapor medium and condensation of it back to the APB. The recondensation presumably occurred before mantle solidification and crust formation on the APB around ~ 4 Myr after CAI formation^18,52^. In addition, this recondensation most likely occurred in the kinetic regime, during which the light K isotope that diffuses faster was preferentially condensed, as opposed to equilibrium condensation that would result in a heavy K isotopic composition in the APB. The condensation process can be approximated by a Rayleigh condensation assuming a CI-like δ^41^K value as the initial vapor composition (δ_0_). Given the extremely low K/U ratio in the APB compared with that in the CI chondrites (Fig. 4a), the degree of vapor condensation is far less than 1%, and hence the fraction of remaining vapor (*F*) is close to unity. Consequently, the δ^41^K value of the APB is essentially determined by the effective fractionation factor (*α’*) according to the equation:

1 $${\delta }^{41}{K}_{{{{{{\rm{condensate}}}}}}}=\alpha ^{\prime} ({\delta }_{0}+1000){F}^{(\alpha ^{\prime} -1)}-1000$$

The *α’* value calculated for the APB varies between 0.9983 and 0.9989, corresponding to an instantaneous isotope fractionation of −1.7 to −1.1‰ between condensed and vapor phases. This *α’* value is similar to that calculated for K condensation into the CB chondrules (0.9984, ref. 29). In contrast, it is significantly reduced from the ideal kinetic fractionation factor (α) of 0.9753 [calculated as √39/41], which corresponds to an instantaneous K isotope fractionation of −25‰ between condensed and vapor phases at infinite supersaturation. The difference between α and *α’* implies 93% to 96% vapor saturation (*P*_*sat*_*/P*) using the equation below:^60^

2 $$\alpha ^{\prime} -1=(\alpha -1)\left(1-\frac{{P}_{{{{{{\rm{sat}}}}}}}}{P}\right)$$

This level of vapor saturation is consistent with 95% saturation estimated for vapor-melt fractionation in a magma-ocean setting for asteroid-sized bodies^10^. Vapor condensation-induced light K isotopic compositions have previously been inferred for chondrules^28,29^ and some lunar non-mare rocks^33^. However, the majority of non-mare rocks remain isotopically heavier than Earth, indicating that condensation has a restricted role on the Moon. In contrast, our study suggests that condensation could be an essential control of volatile element distribution at an asteroid scale. These findings are a step towards the origin of the extremely K-depleted APB. We propose that extensive evaporation and incipient recondensation during the magma-ocean stage provide a straightforward explanation for the strikingly light K isotopic compositions of angrites and their associated characteristics of substantial volatile depletion (e.g., super-chondritic Mn/Na ratios and Mg, Fe, and Si isotope ratios). Nevertheless, the specific physical mechanisms underlying these processes and the possible role of unrecognized processes or planetary reservoirs remain to be explored. Future investigation on a more extensive set of angrites, in particular the recently found dunitic angrite (NWA 8535), is highly desirable for this effort.

The partial recondensation model proposed for the APB would need to be tested with MVEs that are more volatile than K. It is noteworthy, however, that factors other than volatility need to be considered, such as an element’s solid/melt partition coefficient, oxidation state, evaporation congruency, gas species, diffusion coefficient, and activity coefficient^58^. For example, significant amounts of Zn can be accommodated in olivine, spinels, and metal phases; therefore, mantle heterogeneity and magmatic differentiation may complicate the interpretation of Zn isotopic composition of angrites and its comparison with K isotopes. In contrast, Rb behaves similarly to K, and is thus expected to have a light isotopic composition in angrites. The single δ^87^Rb value reported for Sah 99555 (0.12 ± 0.03‰) is similar to CI chondrites (0.19 ± 0.13‰) and terrestrial igneous average (−0.12 ± 0.06‰), but markedly lower than eucrites (up to 1.51‰) that are depleted in Rb due to planetary evaporation^61^. Because this specimen was not cleaned before analyses, it contains significantly more Rb (0.4 ppm, ref. 61) than mineral separates reported for Sah 99555 (0.016–0.073 ppm, ref. 14). Consequently, the authors considered this δ^87^Rb value to mainly reflect crustal contamination that overprinted the original isotopic signature of the sample^61^. Further analyses on precleaned samples are required before a definitive conclusion can be reached. Integrated with future quantitative modeling of the elemental and isotopic compositions of the APB, a clearer picture of its origin will likely emerge.

### Implications for nebula-wide δ^41^K variability

The origin of isotopic variations in the Solar System provides critical constraints on the source and nature of the building blocks of terrestrial bodies. The covariation of δ^41^K with nucleosynthetic ε^54^Cr and ε^64^Ni anomalies in bulk meteorites has been interpreted as evidence for locally inherited nucleosynthetic heterogeneity of their parent bodies from the protosolar molecular cloud^27^. The APB and Vesta have similar ε^54^Cr and µ^48^Ca anomalies^18,62^, suggesting that they were accreted from similar infalling nebular materials or at similar locations in the Solar System (Fig. 4b). However, the δ^41^K value of the APB is ~ 2‰ lower than that of Vesta. This magnitude of isotope variation requires kinetic evaporation (for Vesta) and condensation (for the APB). Conversely, it is too substantial to be explained by presolar heterogeneity given the moderately volatile nature of K. For comparison, the magnitude of nucleosynthetic Zn isotope anomalies is limited to 0.1‰^63,64^. Therefore, the widely divergent δ^41^K signatures of the APB and Vesta indicate that planetary variability in K isotopic composition primarily reflects volatility-dependent fractionations rather than the heterogeneous distribution of presolar nucleosynthetic components.

## Methods

### Potassium isotopic analyses

Chemical and analytical work was carried out at the Institut de Physique du Globe de Paris (IPGP). Given that the upper continental crust contains more than 20,000 ppm K^49^, terrestrial contamination of angrites is inevitable. Radiogenic dating on angrites has shown that terrestrial contamination cannot be removed entirely even after aggressive acid washing^14^, and this procedure may induce mineral dissolution^17^. Since mineral dissolution would result in large K isotope fractionation, we did not leach our angrite samples with acids. As an alternative, we cleaned fresh chips of angrite samples with Milli-Q water in an ultrasonic bath three times for more than 15 minutes per cleaning. As shown in Mittlefehldt et al^51^., this procedure significantly reduced surface contamination from the continental crust. The cleaned angrite chips were then dried and pulverized using an agate mortar.

To effectively separate the vanishingly small amounts of K from the matrix elements, ~62.5–133.9 mg of powdered angrites were dissolved in Savillex screw-top Teflon beakers using sequential addition of concentrated HF-HNO_3_ (3:1), HCl-HNO_3_ (3:1), and HNO_3_. In the HF-HNO_3_ step, the Teflon beakers were capped with Ultem sockets and heated in Analab EvapoClean at 150 °C for five days. After complete dissolution, sample solutions were evaporated to dryness, refluxed with 0.5 mol L^−1^ HNO_3_, and redissolved in 0.5 mol L^−1^ HNO_3_. For chondrites, ~15 mg of sample powders were dissolved in the same way as for angrites. An aliquot of 1 mL seawater was dried down and refluxed successively with concentrated HNO_3_ and 0.5 mol L^−1^ HNO_3_.

Potassium was isolated from the matrix using established protocols adopted from the University of Washington, Seattle^65^. Sample solutions were loaded onto Bio-Rad Poly-Prep columns filled with 2 mL of Bio-Rad AG 50W-X8 cation exchange resin (200–400 mesh). Matrix elements were eluted with 13 mL of 0.5 mol L^−1^ HNO_3_, and the K fraction was collected in the following 22 mL of the same acid. Two passes of column chemistry were performed for terrestrial standards, three for enstatite and ordinary chondrites, and four for carbonaceous chondrites. For angrites, the dissolved solution of a given sample was first split and loaded separately to eight cation-exchange columns, and every four collected K fractions were combined. These two K fractions were then processed through another round of column chemistry, and the collected K fractions were combined into one. Each combined K fraction was processed through six more rounds of column chemistry.

The collected K intervals (22 mL) are much wider than the K peak (~15 mL) to ensure complete recovery^65^. The column yields were checked on Sapphire^TM^ CC-MC-ICP-MS, which are ≥99.3%, consistent with an average K yield of 99.7 ± 1.1% reported previously^65^. Therefore, no significant loss of K occurred during column chemistry. The residual matrices in the purified K fractions are negligible and would not affect K isotopic measurements (Supplementary Fig. 8). The total procedural blank varies between 3.7 ng and 6.2 ng, which is insignificant (≤0.3%) compared with the amounts of K in dissolved angrite solutions (2.1–13.5 µg).

Potassium isotope ratios were measured on a Nu Sapphire^TM^ CC-MC-ICP-MS following the protocol described in Moynier et al^26^. Our previous work^26^ has shown that accurate δ^41^K values can be obtained on this instrument for solutions with a K concentration down to 25 ppb. In this study, sample and K standard solutions were both diluted to 75 ppb with 3% HNO_3_ and introduced into the mass spectrometer by an Apex Omega desolvation nebulizer system, which yielded a total K signal of over 200 V. Instrumental fractionation was corrected by alternating analysis of sample solutions and K Standard Reference Material (SRM 3141a) from the National Institute of Standards and Technology (NIST). Argon-related interferences were removed by reaction with H_2_, and the ion beams of ^41^K^+^ and ^39^K^+^ were measured simultaneously on the peak center in low-mass resolution. A blank of 3% HNO_3_ was measured before each analysis and subtracted from the measured ion beams. The K isotopic composition of each sample is reported as the average of N repeated analyses relative to NIST SRM 3141a in delta notation:

3 $${ \delta }^{41}K(‰)=\left[\left({\,}^{41}K/{\,}^{39}K\right)_{{{{{\rm{sample}}}}}}\Bigg/\left({\,}^{41}K/{\,}^{39}K\right)_{{{{{\rm{NIST \,SRM3141a}}}}}} - 1\right] \times {1000}$$

Analytical uncertainties are reported as both 2 SD (standard deviation) and 95% c.i. (confidence interval). The δ^41^K values of two terrestrial standards and seven chondrite meteorites analyzed in this study agree well with published results, confirming the accuracy of our analyses (Table 1 and Supplementary Fig. 1). In addition, replicate and duplicate analyses yielded consistent results. The full-procedural reproducibility has been evaluated by two dissolutions of a CI chondrite (Orgueil) and two angrites (NWA 12004 and Sah 99555) (see Supplementary Note 3 and Supplementary Fig. 9 for details). Orgueil measured in seven analytical sessions yielded an arithmetic mean δ^41^K of −0.10 ± 0.03‰ (2 SD), and NWA 12004 measured in four analytical sessions yielded an arithmetic mean δ^41^K of −0.44 ± 0.04‰ (2 SD). This level of reproducibility is consistent with the long-term external reproducibility of ± 0.04‰ (2 SD) based on repeated analyses of an international basalt standard BHVO-2^26^.

### Elemental concentration measurements

An aliquot of each acid-dissolved angrite sample was used for elemental concentration measurements with an Agilent 7900 ICP-QMS housed at the IPGP. Another aliquot of the same powdered angrite sample was dissolved using alkali hydroxides to measure Si contents on this instrument. Elemental concentrations were measured in low-resolution mode. Sample solutions were aspirated into a Scott spray chamber using a MicroMist micro-nebulizer (uptake rate = 0.2 mL/min). Elements with atomic masses between 23 (Na) and 75 (As) were measured using a collision-reaction cell supplied with He gas (5 mL/min) to remove polyatomic interferences. Scandium, In, and Re were used as internal standards to correct for signal drift and matrix effects. Count measurements were converted to solution concentrations by analyzing a mixture of certified standards with concentrations spanning the range of the samples. Data are reported in Supplementary Tables 1 and 2.

## Supplementary information

Supplementary Information

## Data Availability

The data generated from this study are provided in Tables 1 and 2 (for K isotopes) and Supplementary Tables 1 and 2 (for elemental concentrations). Referenced data supporting the findings of this study are available in the Supplementary Information files, the published works cited, and the Source Data files. Source data are provided with this paper.

## Source data

Source data
