# Supplementary material for: Potassium isotope heterogeneity in the early Solar System controlled by extensive evaporation and partial recondensation
Source: Nat Commun. 2022 Dec 12;13:7669. doi: 10.1038/s41467-022-35362-7 (PMC9744853; doi:10.1038/s41467-022-35362-7)
Supplement: Supplementary file 1 — Supplementary Information [file 41467_2022_35362_MOESM1_ESM.pdf]

Supplementary Information for

Potassium isotope heterogeneity in the early Solar System controlled by extensive evaporation and partial recondensation

Yan Hu[1*], Frédéric Moynier[1*], Martin Bizzarro[1,2]

Correspondence to: yanhu@ipgp.fr and moynier@ipgp.fr

This PDF file includes:

Supplementary Notes (3)

Supplementary Figures (9)

Supplementary Tables (6)

Supplementary References (53)

Supplementary Note 1

Mineralogy and petrology of studied angrites

Angrites are distinguished from other planetary basalts by their characteristic $\Delta^{17}O$ values of −0.064 ± 0.018‰ (2σ)[1, 2]. Their uniform $\Delta^{17}O$ values have been interpreted as evidence of effective homogenization during an early magma-ocean event on the angrite parent body (APB)[1]. Basaltic angrites, as those analyzed in this study, were formed by melting and differentiation on the APB under oxidizing conditions (with oxygen fugacity of ~ 1 log unit above the iron-wüstite buffer)[3, 4]. The five samples selected for this study are quenched volcanic angrites, which solidified rapidly near the surface of the APB following their eruption. Therefore, they underwent limited crystal fractionation as compared with plutonic angrites (Supplementary Fig. 2). The bulk compositions of Sahara 99555, NWA 12004, NWA 12320, and NWA 7203 represent angrite melts, while NWA 12774 consists of a mixture of melt and olivine crystals. We provide a description of their mineralogy and petrographic characteristics below. More detailed information can be found from the Meteoritical Bulletin Database online and in Keil[4].

Sahara 99555 has a fine-grained ophitic texture, estimated to have a cooling rate of ~ 50ºC/hour[5]. It consists mainly of anorthite (33-37%), Al-Ti clinopyroxene (24-32%), and olivine (30-42%). It shares similarities in mineralogy and petrology to the groundmass of Asuka 881371[5] and is compositionally similar to D'Orbigny[6].

NWA 12004 has a diabasic texture with an average grain size of 0.5 mm[7]. It is mainly composed of calcic olivine, augite, and anorthite ($An_{99.5-99.6}$) with estimated modal abundances of 27%, 37%, and 36%, respectively, based on our CIPW calculation. It bears similarity in many aspects with NWA 12320 and Sahara 99555[7].

NWA 12320 has a diabasic texture with an average grain size of 0.3-0.5 mm[7]. It consists primarily of calcic olivine, Al-Ti-augite, and anorthite ($An_{99.6-99.7}$)[7] with estimated modal abundances of 32%, 32%, and 36%, respectively, based on our CIPW calculation. This angrite contains sparse vesicles with interior coatings of secondary calcite and barite. It has a more heterogeneous composition than other angrites. For example, Irving et al.[7] reported $\Delta^{17}O$ values ranging between −0.063‰ and −0.046‰ for this angrite, which is consistent with those measured in other angrites. By contrast, Rider-Stokes et al.[2] reported $\Delta^{17}O$ values (−0.023‰ to −0.017‰) that are closer to the terrestrial fractionation line ($\Delta^{17}O = 0$). In addition, the aliquot of NWA 12320 we analyzed contains 166.3 ppm Ba, which is twice the concentration (83.1 ppm) of the aliquot analyzed by Irving et al.[7]. The anomalously higher $\Delta^{17}O$ values and Ba concentrations might reflect an unrepresentative sampling of the bulk composition.

NWA 7203 has a very fine-grained (< 10 μm) to medium-grained (~ 1 mm) variolitic texture, which corresponds to a varied cooling rate of ~80ºC/hour for the fine-grained lithologies and ~ 1 ºC/hour for the coarse-grained lithologies[8, 9]. Major minerals include Ca-rich olivine (36-41%) and anorthitic plagioclase ($An_{>99.5}$, 33-37%) with interstitial clinopyroxene (23-30%)[8]. The observed mineral modal abundances are consistent with our estimation based on CIPW calculation (33% olivine, 38% plagioclase, and 29% clinopyroxene). It underwent olivine crystallization from a primary magma compositionally to Sahara 99555 and D'Orbigny[8].

NWA 12774 has a porphyritic texture, characterized by abundant olivine grains embedded in a fine-grained groundmass[10]. The groundmass is composed of anorthite ($An_{99.4-99.7}$), olivine, kirschsteinite, and Al-Ti augite[10]. Our CIPW calculation suggests mineral modal abundances of 51% olivine, 19% clinopyroxene, and 31% anorthite. The olivine-rich nature of NWA 12774 is consistent with its lower rare earth element contents and higher Mg# than the other four angrites analyzed here (Supplementary Fig. 2).

Supplementary Note 2

Extrapolation of K and Rb concentrations in AdoR

Literature data of Rb and Sr concentrations and Rb/Sr ratios reported for AdoR are tabulated in Supplementary Table 3 and plotted in Supplementary Fig. 4. There is a coupled increase in Rb concentration and Rb/Sr ratio from 0.0134 to 0.0829 ppm and from 0.000094 to 0.000497, respectively (Supplementary Fig. 4a). In contrast, Sr concentration data are relatively homogeneous and do not correlate with Rb/Sr ratios (Supplementary Fig. 4b). These observations suggest that the variations in Rb concentration and Rb/Sr ratio are most likely due to sample inhomogeneity rather than crustal contamination, which would result in a coupled increase in Sr concentration. The sample with the highest Rb concentration and Rb/Sr ratio deviates from the linear trend and displays an overabundance of Rb. Excluding this measurement, the average Sr concentration and Rb/Sr ratio in AdoR is 134 ppm and 0.0002377, respectively, which gives an average Rb concentration of 0.0318 ppm. These average values agree well with the dataset reported by Tera et al.[11] and are variably lower than those reported by Wasserburg et al.[12], implying that the subsample of AdoR used in the former study is more representative of the true bulk rock than that used in the latter study. Therefore, the K/Rb ratio of 414.8 calculated from the data reported in Tera et al.[11] is used to extrapolate the K concentration of AdoR. Following Dauphas et al.[13], the K concentration is calculated to be 13.2 ppm based on the multiplication of averaged $(K/Rb)_{AdoR}$, $(Rb/Sr)_{AdoR}$, and $(Sr/U)_{CI}$. This value agrees well with the direct measurement in Tera et al.[11].

Supplementary Note 3

Evaluation of full-procedural reproducibility

The full-procedural reproducibility is evaluated by two dissolutions of a CI chondrite (Orgueil) and two angrites (NWA 12004 and Sah 99555) (Supplementary Fig. 9). Orgueil-1 (13.4 mg) was processed through four rounds of column chemistry, as a typical practice for carbonaceous chondrites. Orgueil-1 measured in five analytical sessions yielded $\delta^{41}K$ values ranging between −0.13 ± 0.03‰ and −0.09 ± 0.06‰. Orgueil-2 (14.7 mg) has been processed through six rounds of column chemistry, as a typical practice for angrites. Measurements of Orgueil-2 in two analytical sessions yielded $\delta^{41}K$ values of −0.10 ± 0.04‰ and −0.08 ± 0.05‰.

The dissolved solution of NWA 12004-1 (123.8 mg) was first split and loaded to eight columns (~ 15 mg equivalent dissolved angrite powder on each column). The eight collected K fractions were split and loaded to two columns during the next round of column chemistry, and these two K fractions were combined into one solution. The combined K fraction then went through six more rounds of column chemistry. Measurements of the purified NWA 12004-1 in two analytical sessions yielded $\delta^{41}K$ values of −0.41 ± 0.04‰ and −0.43 ± 0.02‰. For the second dissolution of this angrite, 62.5 mg of rock powder was used. This dissolution (NWA 12004-2) was first split and loaded to four columns. The four collected K fractions were combined into one and passed through the column six more times. Measurements of the purified NWA 12004-2 in two analytical sessions yielded $\delta^{41}K$ values of −0.46 ± 0.05‰ and −0.45 ± 0.04‰. Finally, two dissolutions of Sah 99555 (125.3 mg and 133.9 mg) were processed in the same way as NWA 12004-1 and other angrites. Sah 99555-1 and Sah 99555-2 yielded consistent $\delta^{41}K$ values of −0.56 ± 0.03‰ and −0.57 ± 0.03‰, respectively.

The seven $\delta^{41}K$ values of Orgueil yielded an arithmetic mean of −0.10 ± 0.03‰ (2SD), and the four $\delta^{41}K$ values of NWA 12004 yielded an arithmetic mean of −0.44 ± 0.04‰ (2SD). This level of reproducibility is consistent with the long-term external reproducibility of ± 0.04‰ (2SD) based on repeated measurements of an international basalt standard BHVO-2[14].

Supplementary Figures

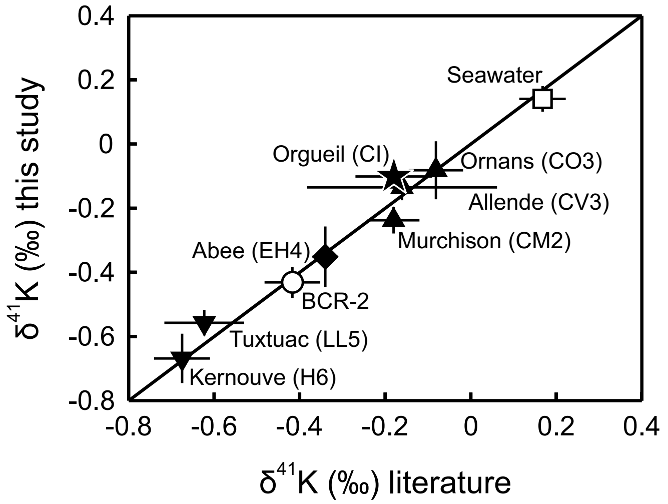

Supplementary Fig. 1 Potassium isotopic compositions of chondrites (filled symbols) and terrestrial standards (open symbols) analysed in this study. Error bars are 2SD. Our results agree well with those reported in Ku and Jacobsen[15] using the same type of instrument, confirming the accuracy of our analyses. Data are reported in Table 1.

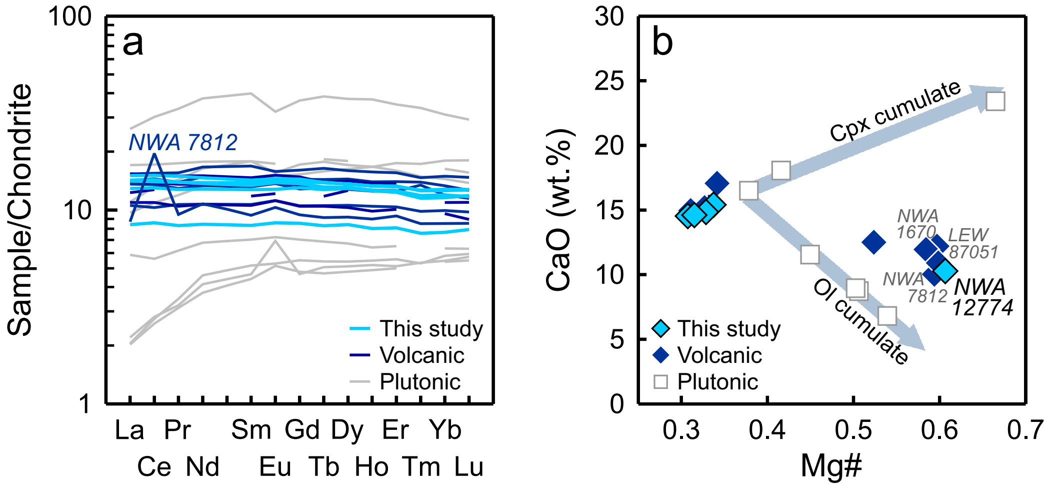

Supplementary Fig. 2 Elemental compositions of bulk angrites analysed in this study. The compositions of volcanic angrites are not significantly affected by mineral fractionation, as supported by **a** their flat, chondritic rare earth element (REE) patterns and **b** restricted variations in CaO content. In contrast, plutonic angrites display strongly fractionated Ca and REE patterns due to extensive crystal fractionation during prolonged cooling underground. The small angrite NWA 7812 (46.2 g) displays a noticeable positive Ce anomaly indicative of aqueous alteration and oxidation of Ce^{3+} to Ce^{4+}, which is less soluble than the trivalent REE[16]. The five samples analysed in this study are compositionally representative of the volcanic angrites documented in the literature. Among them, NWA 12774 has distinctively lower REE contents and higher Mg# [molar ratio of Mg/(Mg + Fe^{2+}) × 100] due to its high olivine content. Similarly high Mg# are reported for NWA 1670[17], NWA 7812[18], and LEW 87051[19]. Literature data for trace elements are from a recent compilation by Tissot et al.[20] and more recent data by Sanborn and Wadhwa[21] and Irving et al.[7, 18]. Literature data for major elements include a compilation by Keil[4] and subsequent publications[7, 10, 18, 22].

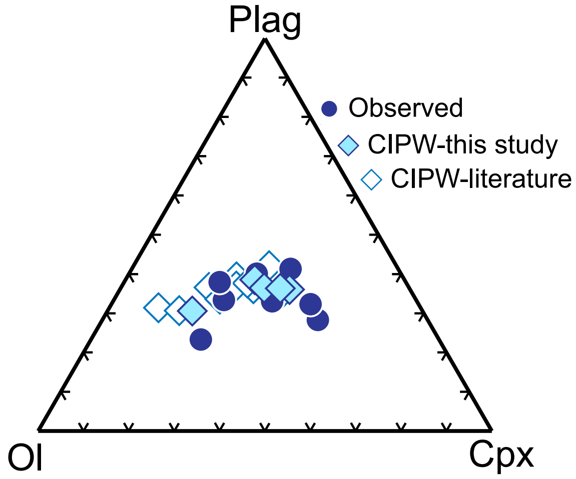

Supplementary Fig. 3 Modal mineralogy of angrites based on petrographic observation and CIPW calculation. The triangle plot shows a subequal proportion of constituent plagioclase (Plag), olivine (Ol), and clinopyroxene (Cpx) in angrites. Filled circle symbols are mineral abundances observed in thin sections that are summarized in Keil[4]. Diamond symbols are normative mineral abundances calculated from major element compositions of angrites. The relative mineral abundances were recalculated to a total of 100%.

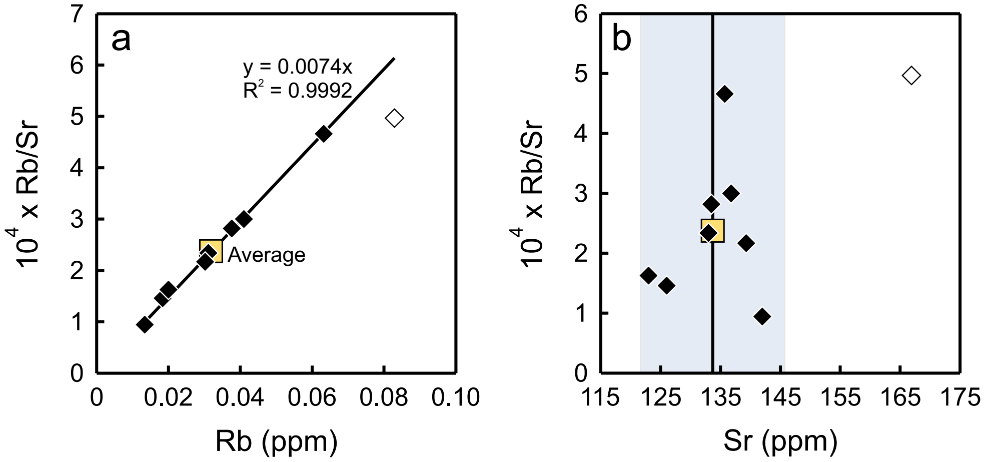

Supplementary Fig. 4 A plot of Rb/Sr ratio versus **a** Rb concentration and **b** Sr concentration of AdoR reported in the literature. The yellow square indicates the calculated average composition. For data sources, please refer to Supplementary Table 3. One pair of measurements from Wasserburg et al.[12] (0.0829 ppm Rb and 167 ppm Sr) deviated from the correlation defined by other data; therefore, these measurements were not included in the average calculation.

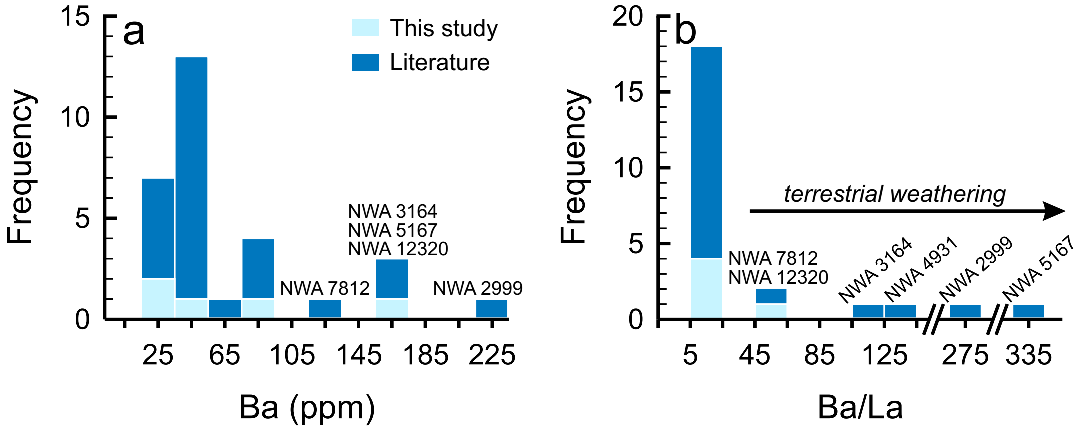

Supplementary Fig. 5 A histogram of **a** Ba concentration and **b** Ba/La ratio in angrites. Elevated Ba concentrations and Ba/La ratios in Saharan meteorites are considered one of the most sensitive indicators of aqueous alteration with resultant sulfate (barite) formation[23, 24]. Except for NWA 12320 analysed in this study, only NWA 2999 with its possible pairs (NWA 4931, NWA 3164, and NWA 5167) and NWA 7812 display Ba concentrations exceeding 95 ppm and Ba/La ratios higher than 25. Chemical weathering of NWA 7812 is indicated by its positive Ce anomaly (Supplementary Fig. 2a). In addition, NWA 2999 and its possible pairs contain various alteration products of metal[21, 25]. They also yielded noble gas ages indicative of terrestrial weathering[26]. Therefore, the elevated Ba concentration and Ba/La ratio in the aliquot of NWA 12320 we analysed possibly reflect the presence of secondary barite due to exposure to salty fluids during terrestrial weathering. This aliquot may thus be an unrepresentative sampling of the true bulk composition of NWA 12320 (see also Supplementary Note 1). The Ba and La concentrations of angrites analysed in this study are reported in Supplementary Table 2. Literature data are compiled from Tera et al.[11], Wasserburg et al.[12], Mittlefehldt and Lindstrom[19], Mittlefehldt[6], Gellissen et al.[27], Riches et al.[28], Irving et al.[7, 18], and Baghdadi et al.[25].

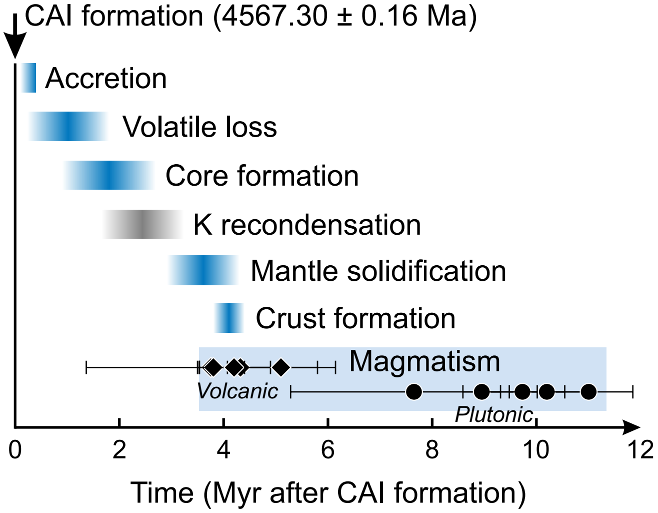

Supplementary Fig. 6 Chronology of the angrite parent body (APB) with its major stages of formation and evolution. The absolute age of CAI formation is from Connelly et al.[29]. Recondensation of K most likely occurred during the early magma-ocean stage on the APB, after radiogenic heating from ^{26}Al decay became trivial (~ 2 Myr after CAI formation, ref. 30) and before mantle solidification and crust formation on the APB (~ 4 Myr after CAI formation, refs. 22 and 33). The accretion timescale of the APB is constrained by Schiller et al.[30]. The timing of volatile loss is based on Rb-Sr dating results of Hans et al.[31] and Sanborn et al.[32]. The timescales of core formation and mantle solidification are taken from Kleine et al.[33]. The timescales of crust formation and magmatism are derived from Zhu et al.[22] and references therein.

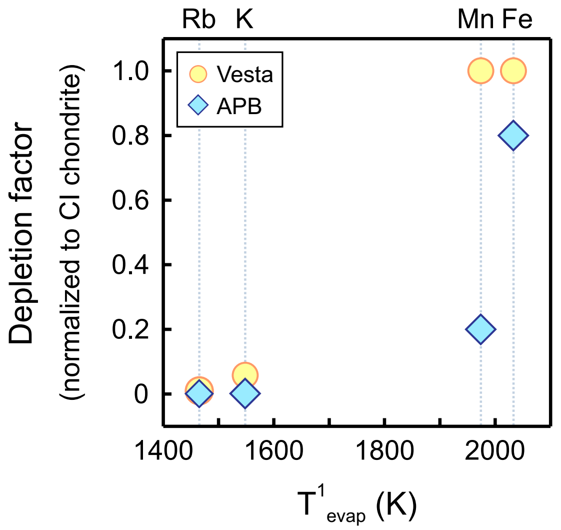

Supplementary Fig. 7 More extensive depletion of volatiles on the APB relative to Vesta. The extents of Mn and Fe depletion are estimated by Sossi et al.[34]. The extents of Rb and K depletion are based on the Rb/Sr and K/U ratios constrained by Dauphas et al.[13] for Vesta and by this study for the APB. The volatility scale shown on the horizontal axis is 1% evaporation temperatures from silicate melt at 1 bar and $\log fO_2 = -10$ taken from Sossi et al.[35]. The equilibrium thermodynamic data for Rb and K were determined experimentally, whereas those for Mn and Fe were based on theoretical calculation. This volatility scale illustrates that K and Rb become significantly more volatile than Mn under oxidizing conditions, which contrasts with their similar 50% condensation temperatures (K: 993 K and Mn: 1123 K)[36] calculated for reducing nebular conditions.

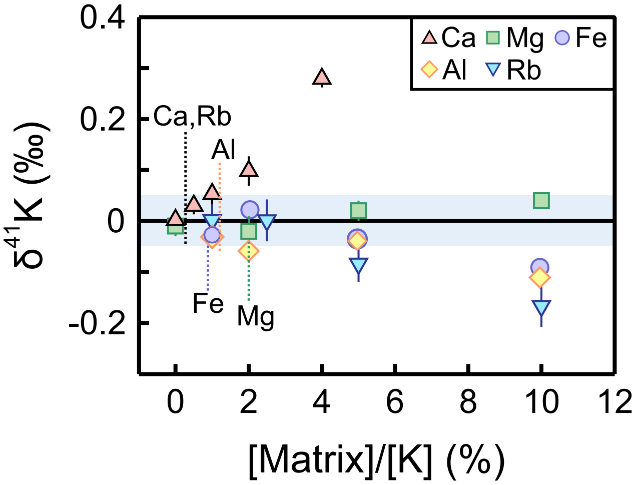

Supplementary Fig. 8 Insignificant amounts of residual matrices in purified K fractions of angrites (as indicated by the vertical dash lines). The blue field represents a typical analytical uncertainty of ± 0.05‰. Error bars are plotted as 95% c.i. When not seen, they are smaller than sample symbols. The matrix effects of Ca and Rb were tested at IPGP, and their presence does not affect K isotopic analyses with [Ca]/[K] ratios up to 1% and [Rb]/[K] ratios up to 2.5%[14]. Previous studies[37, 38] have shown negligible influence of residual Fe, Al, and Mg, with concentration ratios up to 5% for Fe and Al, and up to 20% for Mg. The concentrations of matrix elements in purified angrite solutions were measured on ICP-QMS and reported in Supplementary Table 6. Residues of Na, Ti, Mn, Cr, and V were not detected. The concentration ratios of [Fe]/[K] and [Al]/[K] display a limited range from 0.20 to 0.83% and from 0 to 1.22%, respectively. The concentration ratios of [Mg]/[K] range from 0 to 0.16%, except for a higher ratio of 2.0% in the olivine-rich NWA 12774. The concentration ratios of [Rb]/[K] range from 0.05% to 0.20%. These levels of residual matrices would not affect the K isotopic measurements.

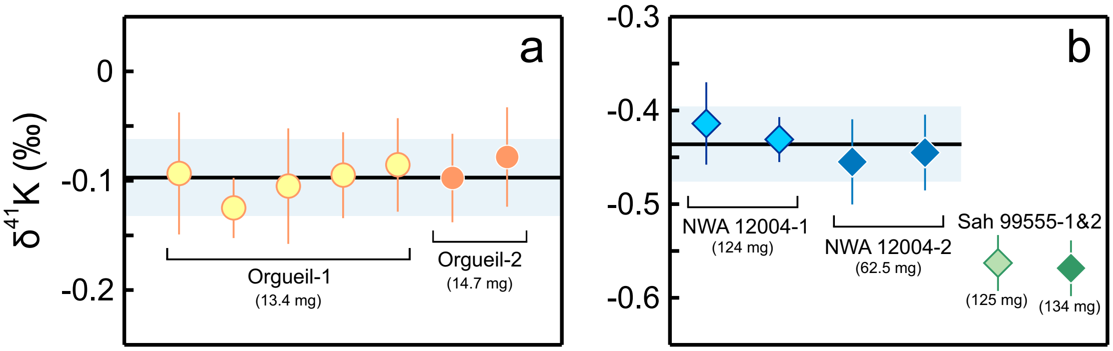

Supplementary Fig. 9 Evaluation of full-procedural reproducibility by two dissolutions of **a** CI chondrite (Orgueil) and **b** two angrites (NWA 12004 and Sah 99555). Each pair of dissolution yielded consistent $\delta^{41}K$ values within uncertainties, validating the accuracy and reproducibility of our analytical methods. The black horizontal lines and the light blue fields represent the average $\delta^{41}K$ value and 2SD, respectively. Error bars are plotted as 95% c.i. Data are reported in Tables 1 and 2.

Supplementary Table 1

Major element compositions (wt.%) of angrites measured in this study.

Sample	NWA 7203	NWA 12320	NWA 12774	NWA 12004	Sah 99555
SiO_2	39.4	40.0	40.4	41.4	40.6
TiO_2	0.81	0.89	0.56	0.89	0.85
Al_2O_3	12.8	12.5	10.5	11.7	12.4
Cr_2O_3	0.07	0.05	0.29	0.04	0.04
FeO_T	23.7	24.8	20.4	24.6	24.2
MnO	0.27	0.27	0.23	0.27	0.26
MgO	6.77	6.81	17.6	6.13	6.24
CaO	15.4	14.9	10.3	14.5	14.6
Na_2O	0.04	0.02	0.01	0.02	0.01
K_2O	0.004	0.007	0.002	0.013	0.005
P_2O_5	0.16	0.17	0.12	0.18	0.17
Total	99.4	100.4	100.4	99.9	99.3
Mg#	33.7	32.9	60.7	30.7	31.5

Element concentrations are measured with an ICP-QMS (ppm) and converted to oxide (wt.%).
Total Fe is reported as FeO_T.
Mg# = 100 × molar Mg/(Mg+Fe).

Supplementary Table 2

Trace element compositions (ppm) of angrites measured in this study.

Sample	NWA 7203	NWA 12320	NWA 12774	NWA 12004	Sah 99555
[Na]	274.0	142.5	102.1	157.7	97.4
[K]	36.7	67.8	16.4	109.1	45.3
[P]	694.5	724.6	545.3	772.4	726.8
[V]	111.8	129.0	105.6	121.7	118.2
[Cr]	447.0	325.7	1984	284.0	247.6
[Mn]	2063	2089	1797	2064	2016
[Co]	26.0	35.2	39.9	35.6	30.7
[Cu]	2.22	3.44	0.27	4.25	3.38
[Zn]	0.44	2.73	0.33	3.20	1.56
[Ga]	1.02	1.18	0.69	1.18	1.08
[Ge]	2.51	2.89	2.25	2.79	2.69
[As]	0.93	1.17	0.33	0.69	1.74
[Rb]	0.08	0.14	0.04	0.23	0.15
[Sr]	98.2	112.0	73.6	102.6	105.5
[Y]	20.7	20.3	11.9	20.6	19.6
[Sb]	0.004	0.012	0.002	0.005	0.012
[Ba]	28.1	166.3	18.9	76.9	37.1
[La]	3.03	3.35	1.98	3.54	3.31
[Ce]	7.80	8.58	5.15	9.04	8.39
[Pr]	1.16	1.26	0.75	1.36	1.26
[Nd]	5.95	6.34	3.92	6.79	6.41
[Sm]	1.94	2.05	1.27	2.18	2.11
[Eu]	0.75	0.81	0.50	0.85	0.82
[Gd]	2.68	2.81	1.76	2.97	2.88
[Tb]	0.48	0.49	0.31	0.52	0.52
[Dy]	3.28	3.32	2.13	3.46	3.46
[Ho]	0.71	0.72	0.45	0.75	0.75
[Er]	2.05	2.08	1.34	2.20	2.19
[Tm]	0.30	0.31	0.20	0.33	0.33
[Yb]	1.94	2.01	1.29	2.11	2.15
[Lu]	0.29	0.29	0.19	0.31	0.31
[Hf]	1.42	1.63	0.98	1.62	1.59
[Th]	0.39	0.44	0.26	0.46	0.44
[U]	0.09	0.11	0.07	0.10	0.10

Supplementary Table 3

Compilation of Rb and Sr concentrations (ppm) and Rb/Sr ratios reported for AdoR in the literature.

Rb (ppm)	Sr (ppm)	Rb/Sr	Reference
0.0311	133	0.000234	Tera et al.[11]
0.0829	167	0.000497	Wasserburg et al.[12]
0.0376	133	0.000282	Wasserburg et al.[12]
0.0632	136	0.000466	Wasserburg et al.[12]
0.0410	137	0.000300	Wasserburg et al.[12]
0.0134	142	0.000094	Lugmair & Galer[39]
0.0302	139	0.000217	Lugmair & Galer[39]
0.0184	126	0.000146	Lugmair & Galer[39]
0.0200	123	0.000163	Riches et al.[28]
	134		Nyquist et al.[40]
0.0319	134	0.000238	Average

Note: the anomalously higher Rb (0.0829 ppm) and Sr concentrations (167 ppm) from the second row were not included in the calculation of the average values.

Supplementary Table 4

Potassium concentrations (ppm) of angrites calculated from literature data on radiogenic ^{40}Ar with an assumed K-Ar age of 4.5 Ga.

Sample	Radiogenic ^{40}Ar (10^{-8} cm^3/g)	$[K]_{adopted}$ (ppm)	K-Ar age (Ga)	$[K]_{calculated}$ (ppm)	Pb-Pb age (Ma)	Reference for radiogenic ^{40}Ar	Reference for $[K]_{adopted}$	Reference for Pb-Pb age
AdoR	50			6.4	4556.45	Munk[41]		Tissot et al.[20]
AdoR	86	64	1.93	11.0		Muller & Zahringer[42]	Muller & Zahringer[42]	
AdoR	61	64	1.55	7.8		Ganapathy & Anders[43]	Muller & Zahringer[42]	
LEW 86010	127	350	0.75	16.3	4558.07	Eugster et al.[44]	Warren & Kallemeyn[45]	Lugmair & Galer[39]; Amelin[46]
LEW 87051	58	<113	<0.99	7.4		Eugster et al.[44]	Warren & Kallemeyn[45]	
Sah 99555	63	47	1.93	8.1	4563.93	Busemann et al.[47]	Bischoff et al.[48]; Mittlefehldt et al.[6]	Tissot et al.[20]
Sah 99555	58	50	1.76	7.4		Bischoff et al.[48]	Bischoff et al.[48]	
D'Orbigny	8	10	1.37	1.0	4563.51	Busemann et al.[47]	Garrison & Bogard[49]	Tissot et al.[20]
D'Orbigny glass	149	31	3.72	19.2		Busemann et al.[47]	Varela et al.[50]; Floss et al.[16]	
NWA 7812	276	80	3.21	35.4		Wieler et al.[51]	Irving et al.[18]	
replicate	376	80	3.68	48.3				

$[K]_{adopted}$ refers to K concentrations adopted by the same authors that measured the radiogenic ^{40}Ar value of a given angrite to derive its K-Ar age.
$[K]_{calculated}$ refers to K concentrations calculated in this study to bring up the K-Ar age of a given angrite to 4.5 Ga based on its published radiogenic ^{40}Ar value.
Note that NWA 7812 has an evidently higher radiogenic ^{40}Ar. This sample also display a strong positive Ce anomaly indicative of aqueous alteration (Supplementary Fig. 2a); therefore, its calculated K concentration is considered unreliable.

Supplementary Table 5

Compiled Sr and U concentrations (ppm) for angrites analysed in this study and in the literature.

Volcanic	Sah 99555	D'Orbigny	NWA 12004	NWA 12320	A 881371	NWA 1296	NWA 1670	LEW 87051	NWA 7203	NWA 12774
Average Sr	106	103	106	112	79.5	104	92.9	80	98.2	73.6
Individual value and reference	107[6], 93.9[28], 117[25], 106 (this study)	126, 142[6], 96, 110[28], 42[32]	110[7], 103 (this study)	111[7], 112 (this study)	79.5[28]	101[28], 107[25]	92.9[25]	67[6], 93[45]	This study	This study
Average U	0.101	0.089	0.097	0.109	0.082	0.069	0.075		0.091	0.069
Individual value and reference	0.106[28], 0.097[25], 0.101 (this study)	0.116[6], 0.081[20], 0.082, 0.077[28]	This study	This study	0.082[28]	0.067[28], 0.07[25]	0.075[25]		This study	This study

Plutonic	NWA 4590	NWA 4801	LEW 86010	NWA 2999	NWA 4931	NWA 6291	NWA 3164	NWA 5167
Average Sr	122	104	120	109	63.9		78.2	56.5
Individual value and reference	133[28], 111[32]	109, 100[28], 168[32]	120[52]	109[32]	63.9[28]		78.2[25]	56.5[25]
Average U	0.111	0.082	0.15	0.13	0.032	0.139	0.337	0.019
Individual value and reference	0.096[28], 0.126[20]	0.094, 0.081[28], 0.07[20]	0.15[52]	0.13[27]	0.032[28]	0.139[20]	0.337[25]	0.019[25]

Note: NWA 2999 is paired with NWA 4931, NWA 6291, and possibly with NWA 3164 and NWA 5167. However, NWA 4931 and NWA 3164 have considerably different U concentrations from those in NWA 2999 and NWA 6291. Therefore, their U concentrations are not included in the average calculation. Also excluded are the Sr and U concentrations of weathered and metamorphosed NWA 5167[25], which are considerably lower than NWA 2999 and paired angrites.

The average Sr and U concentrations calculated from basaltic angrites are 98.3 ppm and 0.100 ppm, respectively. Assuming that angrites have a Sr/U ratio identical to that of CI chondrite (1004 from Barrat et al.[53]), the calculated average U concentration of angrites is 0.098 ppm.

Supplementary Table 6

Concentration ratios of matrix element relative to K in purified K fractions of angrites.

Sample	Na/K	Rb/K	Ca/K	Mg/K	Fe/K	Al/K	Ti/K	Mn/K	Cr/K	V/K
NWA 12004	0	0.07%	0.04%	0.08%	0.28%	0	0	0	0	0
NWA 12774	0	0.05%	0.23%	2.00%	0.83%	0.67%	0	0	0	0
Sah 99555	0	0.20%	0.14%	0.16%	0.24%	0.04%	0	0	0	0.001%
NWA 7203	0	0.08%	0.09%	0	0.20%	1.22%	0	0	0	0
NWA 12320	0	0.10%	0.06%	0.07%	0.36%	0.40%	0	0	0	0.002%

Supplementary References

1. Greenwood RC, Franchi IA, Jambon A, Buchanan PC. Widespread magma oceans on asteroidal bodies in the early Solar System. *Nature* **435**, 916-918 (2005).
2. Rider-Stokes B, Greenwood R, Anand M, Franchi I, White L. Revising the angrite fractionation line: new insights from high-precision oxygen isotope studies (abstract). *Annu mtg Meteorit Soc* LPI Contrib. No. **2609**, 6071 (2021). https://ui.adsabs.harvard.edu/abs/2021LPICo2609.6071R/abstract.
3. McKay G, Le L, Wagstaff J, Crozaz G. Experimental partitioning of rare earth elements and strontium: Constraints on petrogenesis and redox conditions during crystallization of Antarctic angrite Lewis Cliff 86010. *Geochim Cosmochim Acta* **58**, 2911-2919 (1994).
4. Keil K. Angrites, a small but diverse suite of ancient, silica-undersaturated volcanic-plutonic mafic meteorites, and the history of their parent asteroid. *Geochem* **72**, 191-218 (2012).
5. Mikouchi T, McKay G, Le L, Mittlefehldt D. Preliminary examination of Sahara 99555: Mineralogy and experimental studies of a new angrite (abstract). No. JSC-CN-6017. *Lunar Planet Sci* **31** (2000). https://ntrs.nasa.gov/citations/20000040790.
6. Mittlefehldt DW, Killgore M, Lee MT. Petrology and geochemistry of D'Orbigny, geochemistry of Sahara 99555, and the origin of angrites. *Meteorit Planet Sci* **37**, 345-369 (2002).
7. Irving A, et al. Petrologic, elemental, isotopic, and magnetic characterization of vesicular hypabyssal angrites Northwest Africa 12004 and Northwest Africa 12320 (abstract). *Lunar Planet Sci* LPI Contrib. No. **2132**, 2758 (2019). https://www.hou.usra.edu/meetings/lpsc2019/pdf/2758.pdf.
8. Mikouchi T, Bizzarro M. Mineralogy and petrology of NWA 7203: A new quenched angrite similar to NWA 1296 and NWA 1670 (abstract). *Meteorit Planet Sci* **75** (Suppl), 5120 (2012). https://www.lpi.usra.edu/meetings/metsoc2012/pdf/5120.pdf.
9. Hayashi H, et al. Unique igneous textures and shock metamorphism of the Northwest Africa 7203 angrite: Implications for crystallization processes and the evolutionary history of the angrite parent body. *Meteorit Planet Sci* **57**, 105-121 (2022).
10. Irving A, Carpenter P, Tepper J, Righter M, Lapen T, Busemann H. Petrology and chemical composition of olivine-pyroxene-phyric quenched angrite Northwest Africa 12774 (abstract). *Lunar Planet Sci* 2399 (2020). https://www.hou.usra.edu/meetings/lpsc2020/pdf/2399.pdf.
11. Tera F, Eugster O, Burnett D, Wasserburg G. Comparative study of Li, Na, K, Rb, Cs, Ca, Sr and Ba abundances in achondrites and in Apollo 11 lunar samples. *Geochimica et Cosmochimica Acta* **1** (Suppl), 1637 (1970). https://ui.adsabs.harvard.edu/abs/1970GeCAS...1.1637T/abstract.
12. Wasserburg GJ, Tera F, Papanastassiou DA, Huneke JC. Isotopic and chemical investigations on Angra dos Reis. *Earth Planet Sci Lett* **35**, 294-316 (1977).
13. Dauphas N, et al. The extent, nature, and origin of K and Rb depletions and isotopic fractionations in Earth, the Moon, and other planetary bodies. *Planet Sci J* **3**, 29 (2022).

14. Moynier F, et al. Potassium isotopic composition of various samples using a dual-path collision cell-capable multiple-collector inductively coupled plasma mass spectrometer, Nu instruments Sapphire. *Chem Geol* **571**, 120144 (2021).

15. Ku Y, Jacobsen SB. Potassium isotope anomalies in meteorites inherited from the protosolar molecular cloud. *Sci Adv* **6**, eabd0511 (2020).

16. Floss C, Crozaz G, McKay G, Mikouchi T, Killgore M. Petrogenesis of angrites. *Geochim Cosmochim Acta* **67**, 4775-4789 (2003).

17. Jambon A, et al. Mineralogy and petrology of the angrite Northwest Africa 1296. *Meteorit Planet Sci* **40**, 361-375 (2005).

18. Irving A, Kuehner S, Chen G, Conrey R, Ziegler K, Pitt D. Petrologic, elemental and oxygen isotopic characterization of Mg-rich, olivine-phyric quenched angrite Northwest Africa 7812 (abstract). *Meteorit Planet Sci* **76** (Suppl), 5249 (2013). http://www.hou.usra.edu/meetings/metsoc2013/pdf/5249.pdf.

19. Mittlefehldt DW, Lindstrom MM. Geochemistry and genesis of the angrites. *Geochim Cosmochim Acta* **54**, 3209-3218 (1990).

20. Tissot FLH, Dauphas N, Grove TL. Distinct $^{238}U/^{235}U$ ratios and REE patterns in plutonic and volcanic angrites: Geochronologic implications and evidence for U isotope fractionation during magmatic processes. *Geochim Cosmochim Acta* **213**, 593-617 (2017).

21. Sanborn ME, Wadhwa M. Trace element geochemistry of coarse-grained angrites from Northwest Africa: Implications for their petrogenesis on the angrite parent body. *Meteorit Planet Sci* **56**, 482-499 (2021).

22. Zhu K, Moynier F, Wielandt D, Larsen KK, Barrat J-A, Bizzarro M. Timing and origin of the angrite parent body inferred from Cr isotopes. *Astrophys J* **877**, L13 (2019).

23. Barrat JA, Blichert-Toft J, Nesbitt RW, Keller F. Bulk chemistry of Saharan shergottite Dar al Gani 476. *Meteorit Planet Sci* **36**, 23-29 (2001).

24. Crozaz G, Floss C, Wadhwa M. Chemical alteration and REE mobilization in meteorites from hot and cold deserts. *Geochim Cosmochim Acta* **67**, 4727-4741 (2003).

25. Baghdadi B, Jambon A, Barrat J-A. Metamorphic angrite Northwest Africa 3164/5167 compared to magmatic angrites. *Geochim Cosmochim Acta* **168**, 1-21 (2015).

26. Nakashima D, Nagao K, Irving AJ. Noble gases in angrites Northwest Africa 1296, 2999/4931, 4590, and 4801: Evolution history inferred from noble gas signatures. *Meteorit Planet Sci* **53**, 952-972 (2018).

27. Gellissen M, Palme H, Korotev R, Irving A. NWA 2999, a unique angrite with a large chondritic component (abstract). *Lunar Planet Sci* 1612 (2007). https://ui.adsabs.harvard.edu/abs/2007LPI....38.1612G/abstract.

28. Riches AJV, et al. Rhenium–osmium isotope and highly-siderophile-element abundance systematics of angrite meteorites. *Earth Planet Sci Lett* **353-354**, 208-218 (2012).

29. Connelly JN, Bizzarro M, Krot AN, Nordlund Å, Wielandt D, Ivanova MA. The absolute chronology and thermal processing of solids in the solar protoplanetary disk. *Science* **338**, 651-655 (2012).

30. Schiller M, Connelly JN, Glad AC, Mikouchi T, Bizzarro M. Early accretion of protoplanets inferred from a reduced inner solar system ^{26}Al inventory. *Earth Planet Sci Lett* **420**, 45-54 (2015).

31. Hans U, Kleine T, Bourdon B. Rb–Sr chronology of volatile depletion in differentiated protoplanets: BABI, ADOR and ALL revisited. *Earth Planet Sci Lett* **374**, 204-214 (2013).

32. Sanborn ME, Carlson RW, Wadhwa M. ^{147,146}Sm–^{143,142}Nd, ^{176}Lu–^{176}Hf, and ^{87}Rb–^{87}Sr systematics in the angrites: Implications for chronology and processes on the angrite parent body. *Geochim Cosmochim Acta* **171**, 80-99 (2015).

33. Kleine T, Hans U, Irving AJ, Bourdon B. Chronology of the angrite parent body and implications for core formation in protoplanets. *Geochim Cosmochim Acta* **84**, 186-203 (2012).

34. Sossi PA, Nebel O, Anand M, Poitrasson F. On the iron isotope composition of Mars and volatile depletion in the terrestrial planets. *Earth Planet Sci Lett* **449**, 360-371 (2016).

35. Sossi PA, Klemme S, O'Neill HSC, Berndt J, Moynier F. Evaporation of moderately volatile elements from silicate melts: experiments and theory. *Geochim Cosmochim Acta* **260**, 204-231 (2019).

36. Wood BJ, Smythe DJ, Harrison T. The condensation temperatures of the elements: A reappraisal. *Am Min* **104**, 844-856 (2019).

37. Chen H, Saunders NJ, Jerram M, Halliday AN. High-precision potassium isotopic measurements by collision cell equipped MC-ICPMS. *Chem Geol* **578**, 120281 (2021).

38. Zheng X-Y, Chen X-Y, Ding W, Zhang Y, Charin S, Gérard Y. High precision analysis of stable potassium (K) isotopes by the collision cell MC-ICP-MS "Sapphire" and a correction method for concentration mismatch. *J Anal At Spectrom* **37**, 1273-1287 (2022).

39. Lugmair GW, Galer SJG. Age and isotopic relationships among the angrites Lewis Cliff 86010 and Angra dos Reis. *Geochim Cosmochim Acta* **56**, 1673-1694 (1992).

40. Nyquist LE, Bansal B, Wiesmann H, Shih C-Y. Neodymium, strontium and chromium isotopic studies of the LEW86010 and Angra dos Reis meteorites and the chronology of the angrite parent body. *Meteoritics* **29**, 872-885 (1994).

41. Munk MN. Argon, krypton, and xenon in Angra dos Reis, Nuevo Laredo, and Norton County achondrites: The case for two types of fission xenon in achondrites. *Earth Planet Sci Lett* **3**, 457-465 (1967).

42. Müller HW, Zähringer J. Rare Gases in Stony Meteorites. In: Millman, PM (eds) Meteor Res. Astrophys Space Sci Lib, vol **12**. Springer, Dordrecht (1969). https://doi.org/10.1007/978-94-010-3411-1_67.

43. Ganapathy R, Anders E. Ages of calcium-rich achondrites—II. Howardites, nakhlites, and the Angra dos Reis angrite. *Geochim Cosmochim Acta* **33**, 775-787 (1969).

44. Eugster O, Michel T, Niedermann S. ^{244}Pu-Xe formation and gas retention age, exposure history, and terrestrial age of angrites LEW86010 and LEW87051: Comparison with Angra dos Reis. *Geochim Cosmochim Acta* **55**, 2957-2964 (1991).

45. Warren P, Kallemeyn G. Geochemistry of the LEW87051 angrite and other basaltic achondrites (abstract). *Lunar Planet Sci* **21**, 1295-1296 (1990). https://adsabs.harvard.edu/full/1990LPI....21.1295W.

46. Amelin Y. U–Pb ages of angrites. *Geochim Cosmochim Acta* **72**, 221-232 (2008).

47. Busemann H, Lorenzetti S, Eugster O. Noble gases in D'Orbigny, Sahara 99555 and D'Orbigny glass—Evidence for early planetary processing on the angrite parent body. *Geochim Cosmochim Acta* **70**, 5403-5425 (2006).

48. Bischoff A, et al. Mineralogy, chemistry, noble gases, and oxygen-and magnesium-isotopic compositions of the angrite Sahara 99555 (abstract). *Meteorit Planet Sci* **35** (Suppl), A27 (2000). http://www.lpi.usra.edu/meetings/metsoc2000/pdf/5071.pdf.

49. Garrison D, Bogard D. ^{39}Ar-^{40}Ar Dating of two angrites and two brachnites (abstract). *Lunar Planet Sci* **34**, 1069 (2003). https://www.lpi.usra.edu/meetings/lpsc2003/pdf/1069.pdf.

50. Varela ME, et al. Glasses in the D'Orbigny angrite. *Geochim Cosmochim Acta* **67**, 5027-5046 (2003).

51. Wieler R, et al. Noble gases in 18 Martian meteorites and angrite Northwest Africa 7812—Exposure ages, trapped gases, and a re-evaluation of the evidence for solar cosmic ray-produced neon in shergottites and other achondrites. *Meteorit Planet Sci* **51**, 407-428 (2016).

52. Kallemeyn G, Warren P. Geochemistry of the LEW86010 angrite (abstract). *Lunar Planet Sci* 20, 496-497 (1989). https://ui.adsabs.harvard.edu/abs/1989LPI....20..496K/abstract.

53. Barrat JA, Zanda B, Moynier F, Bollinger C, Liorzou C, Bayon G. Geochemistry of CI chondrites: Major and trace elements, and Cu and Zn Isotopes. *Geochim Cosmochim Acta* **83**, 79-92 (2012).
